# Supplementary material for: An Unprecedented Tridentate-Bridging Coordination Mode of Permanganate Ions: The Synthesis of an Anionic Coordination Polymer—[CoIII(NH3)6]n[(K(κ1-Cl)2(μ2,2′,2″-(κ3-O,O′,O″-MnO4)2)n∞]—Containing Potassium Central Ion and Chlorido and Permanganato Ligands
Source: Molecules. 2024 Sep 19;29(18):4443. doi: 10.3390/molecules29184443 (PMC11433707; doi:10.3390/molecules29184443)
Supplement: Supplementary file 1 [file molecules-29-04443-s001.zip › molecules-3091837-supplementary.pdf]

Electronic Supplementary Material

An unprecedented tridentate-bridging coordination mode of permanganate-ion: The synthesis of an anionic coordination polymer - $[Co^{III}(NH_3)_6]_n[(K(\kappa^1\text{-}Cl)_2(\mu^{2,2',2''}\text{-}(\kappa^3\text{-}O,O',O''\text{-}MnO_4)_2)_n{}^{\infty}]$ -containing potassium central ion and chlorido and permanganato ligands

László Kótai[1*], Kende Attila Béres[1,2], Attila Farkas[3], Berta Barta Holló[4], Vladimir M. Petrusevski[5], Zoltán Homonnay[1,6], László Trif[1], and Laura Bereczki[1]

[1]Institute of Materials and Environmental Chemistry, HUN-REN Research Centre for Natural Sciences, Budapest, Hungary; beres.kende.attila@ttk.hu (K.A.B.); nagyne.bereczki.laura@ttk.mta.hu (L.B.)
[2]György Hevesy PhD School of Chemistry, ELTE Eötvös Loránd University, Budapest, Hungary
[3]Department of Organic Chemistry and Technology, Faculty of Chemical Technology and Biotechnology, Budapest University of Technology and Economics, Budapest, Hungary; farkas.attila@vbk.bme.hu
[4]Department of Chemistry, Biochemistry and Environmental Protection, Faculty of Sciences, University of Novi Sad, Novi Sad, Serbia
[5]Faculty of Natural Sciences and Mathematics, Ss. Cyril and Methodius University, MK-1000 Skopje, North Macedonia; vladop@pmf.ukim.mk
[6]Institute of Chemistry, ELTE Eötvös Loránd University, Budapest, Hungary; homonnay.zoltan@ttk.elte.hu
*Correspondence: kotai.laszlo@ttk.hu

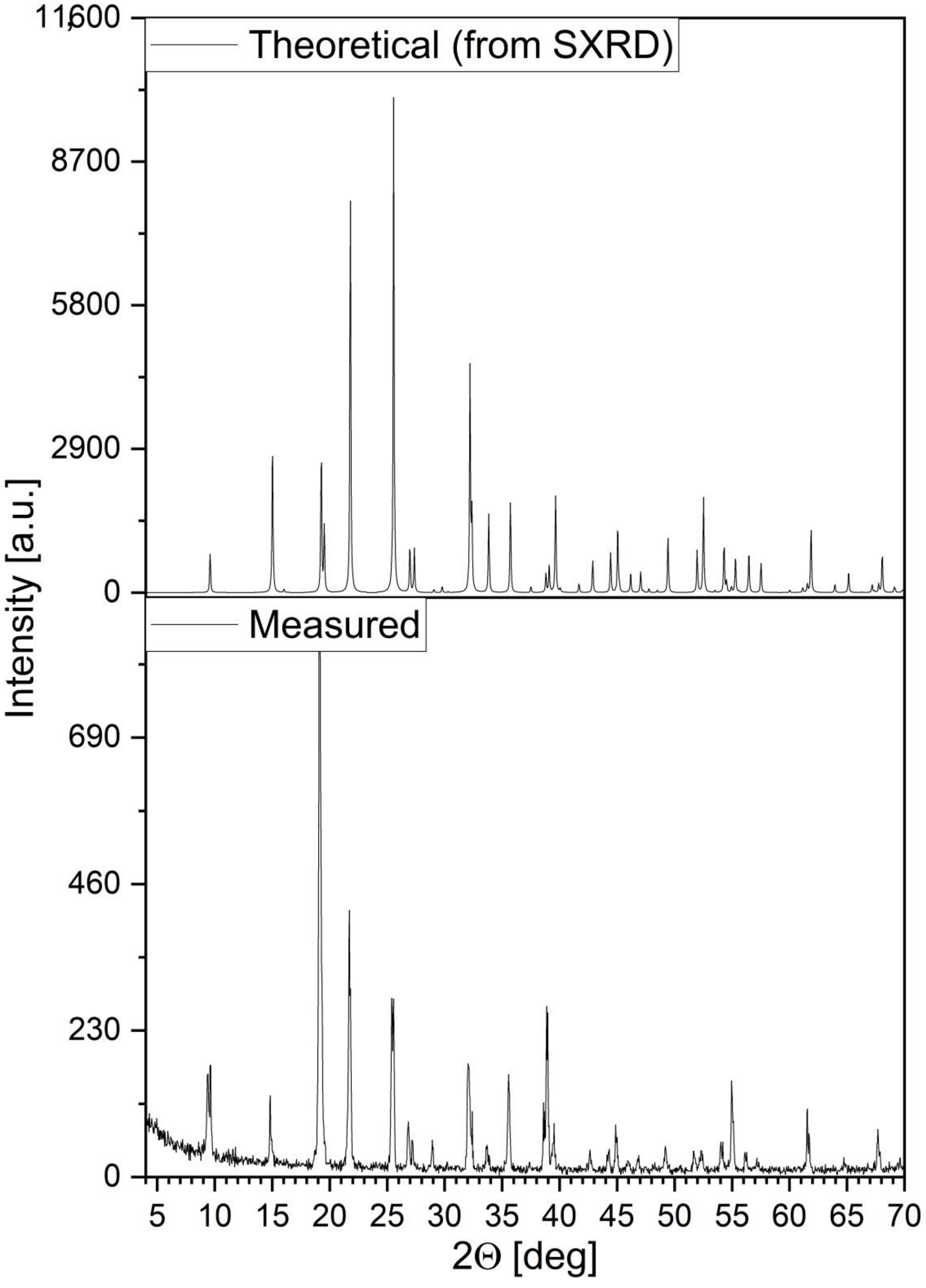

Figure S1. The theoretical (100 K) and experimental (298 K) powder X-ray diffractogram of compound **1** at room temperature.

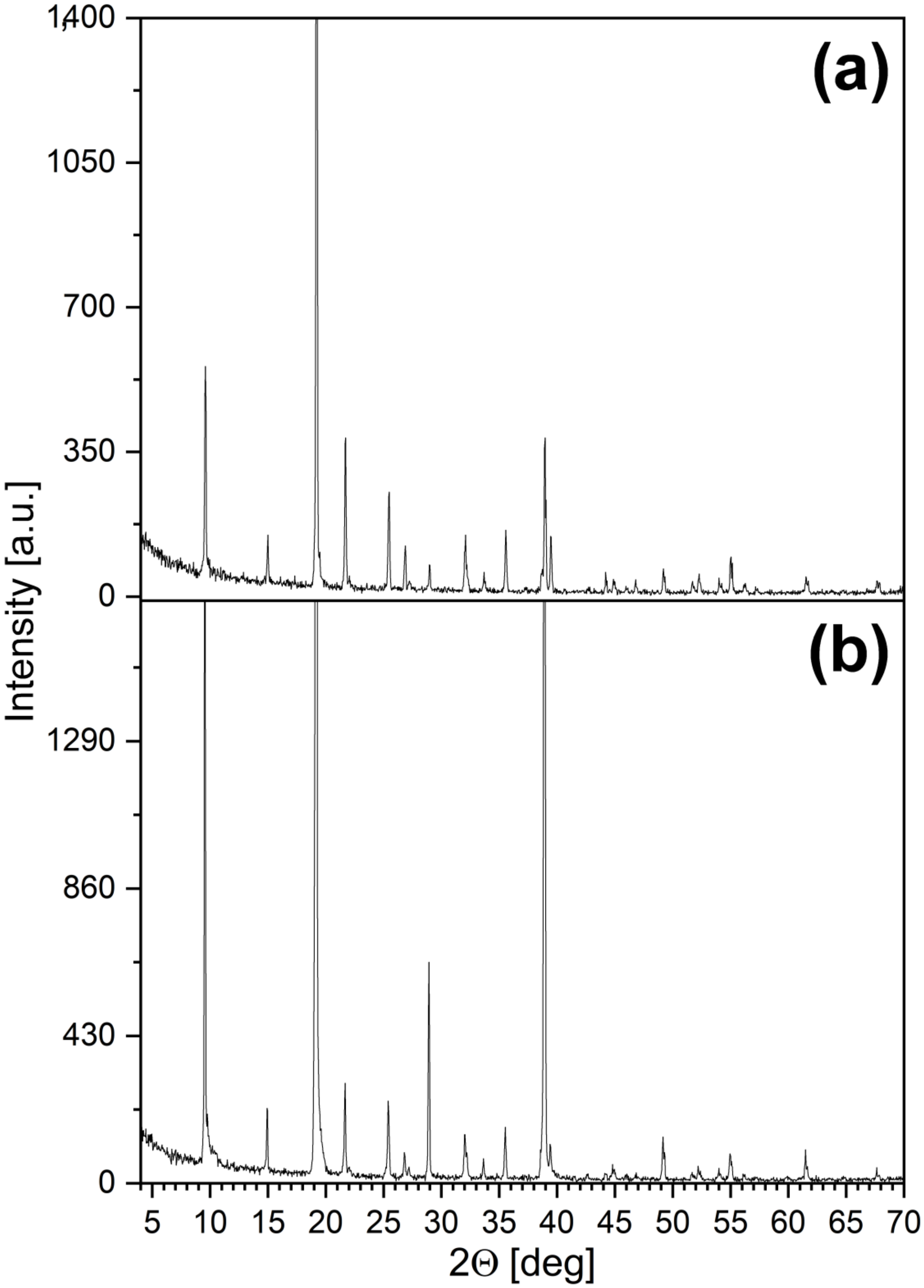

Figure S2. The experimental powder X-ray diffractogram of compound **1** was prepared at (a) room temperature and (b) at 5 °C.

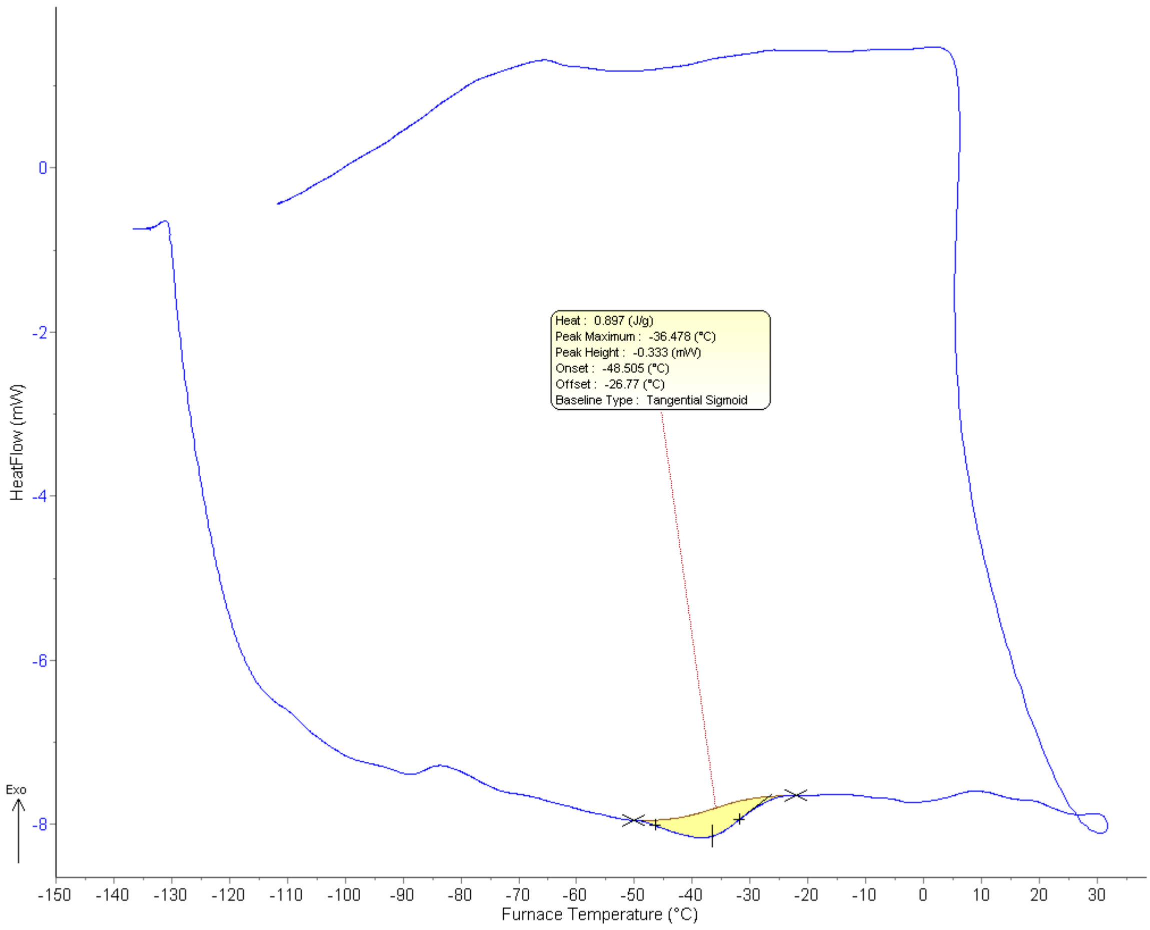

Figure S3. The cryo-DSC curve of compound **1**.

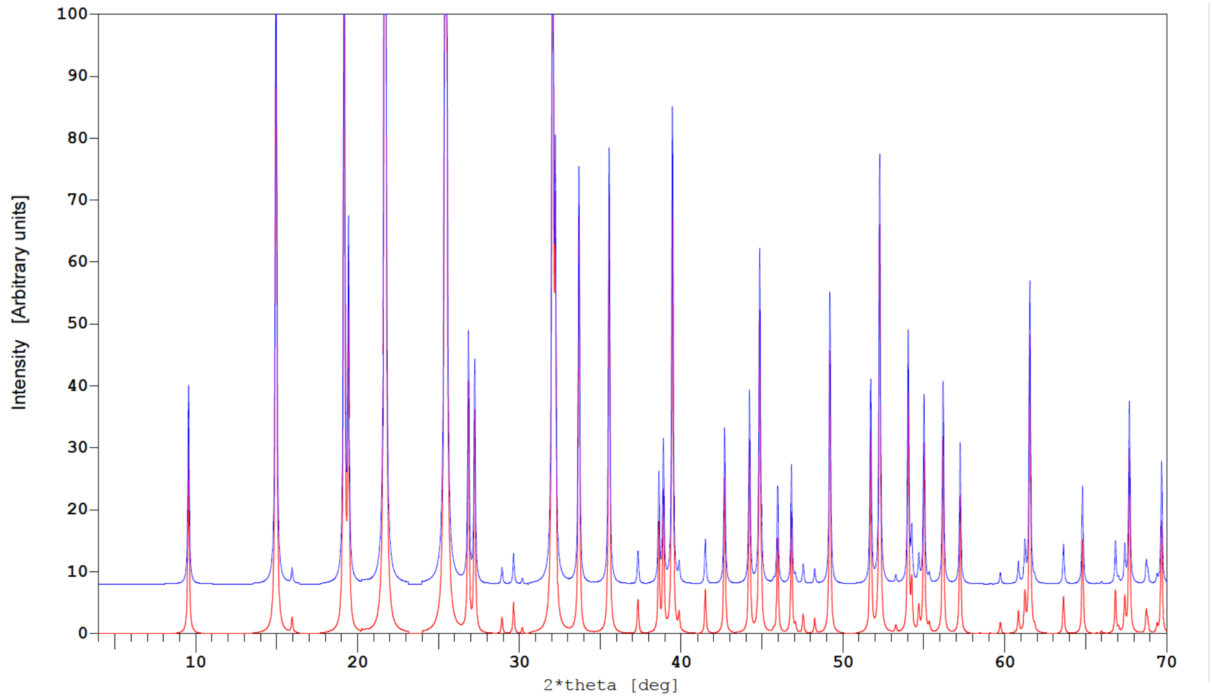

Figure S4. The comparison of the theoretical PXRDs of compound **1** (blue-100 K, red-273K).

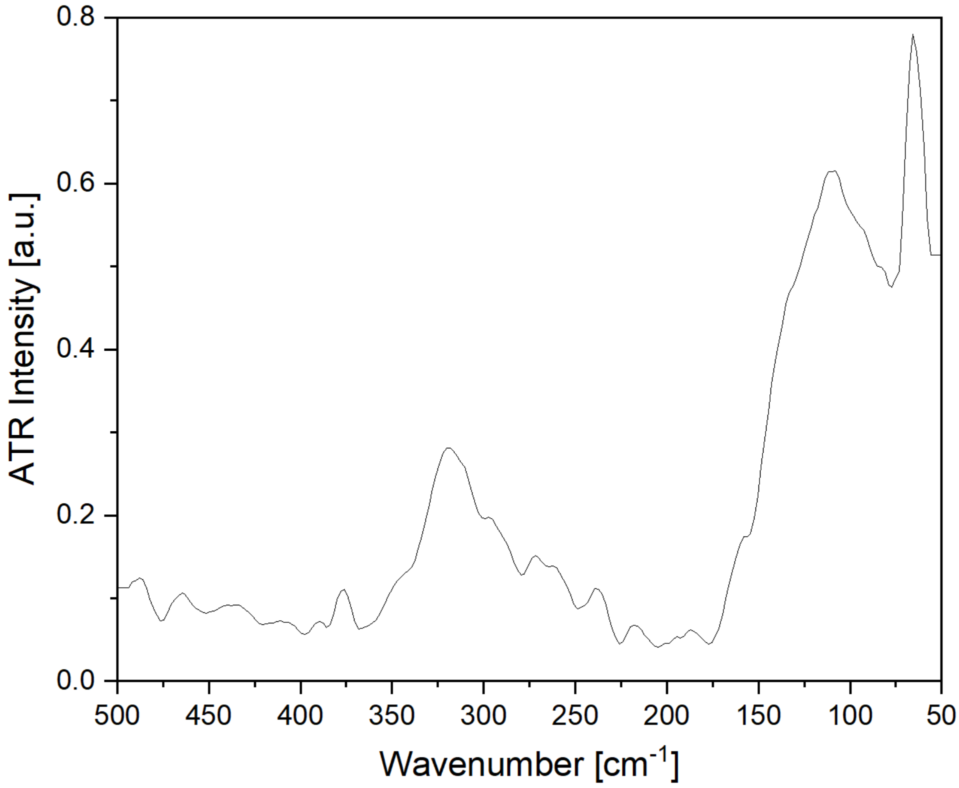

Figure S5. Far-IR spectrum of compound **1**.

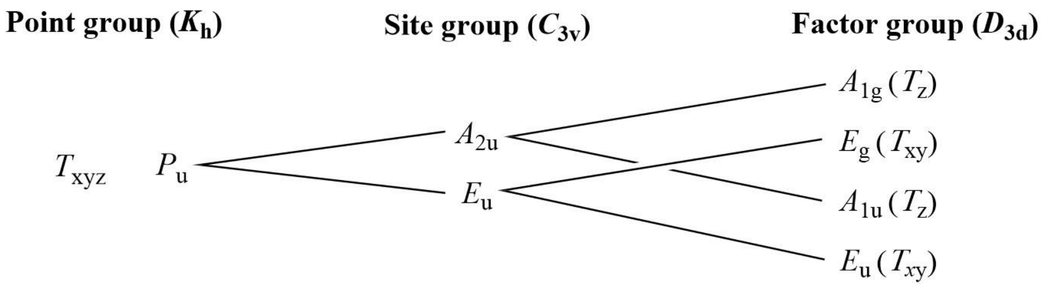

Figure S6. The correlation analysis of the chloride-ion translation modes in compound **1**.

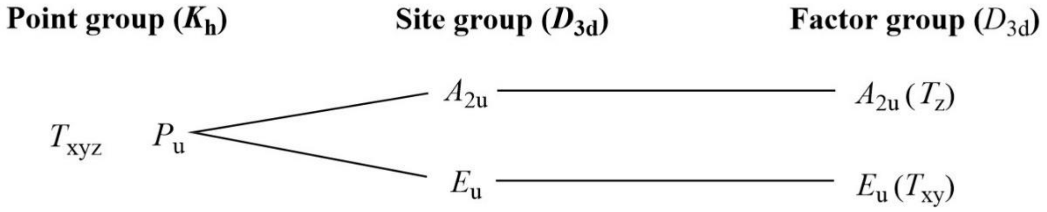

Figure S7. The correlation analyses of the central K^+ and Co^{III} ions in compound **1**.

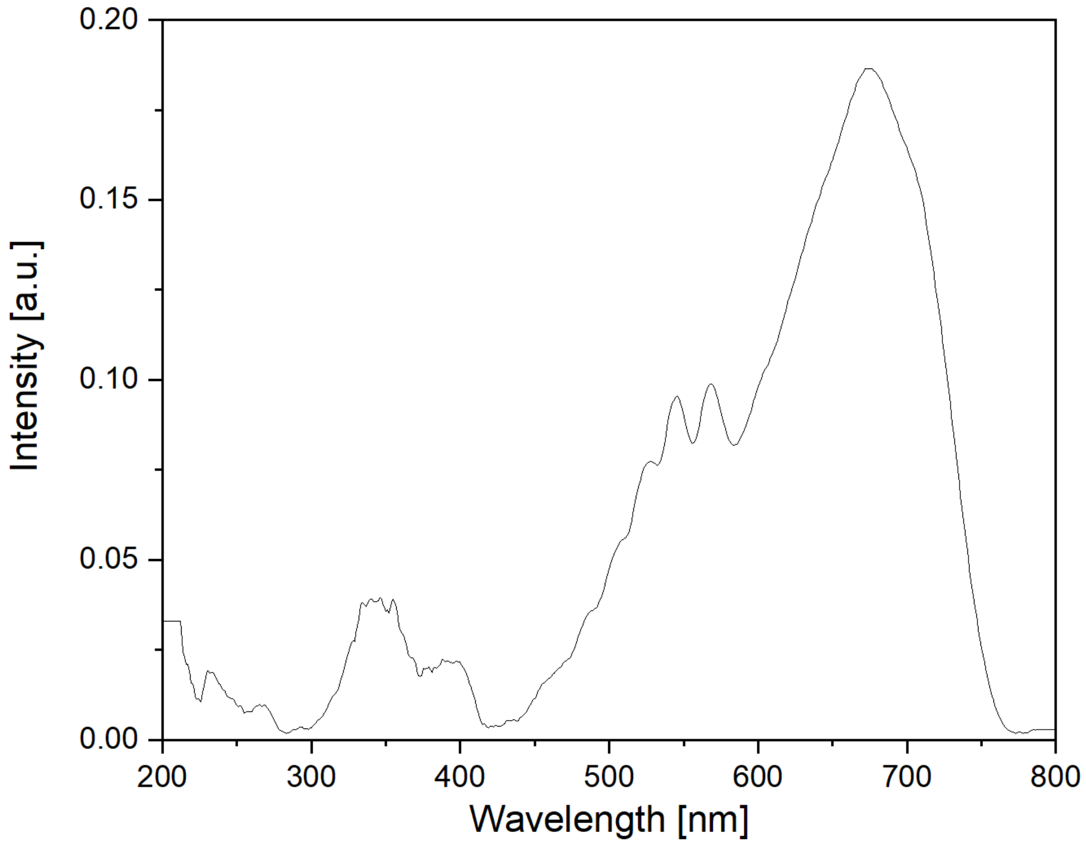

Figure S8. The UV-VIS spectrum of compound **1**.

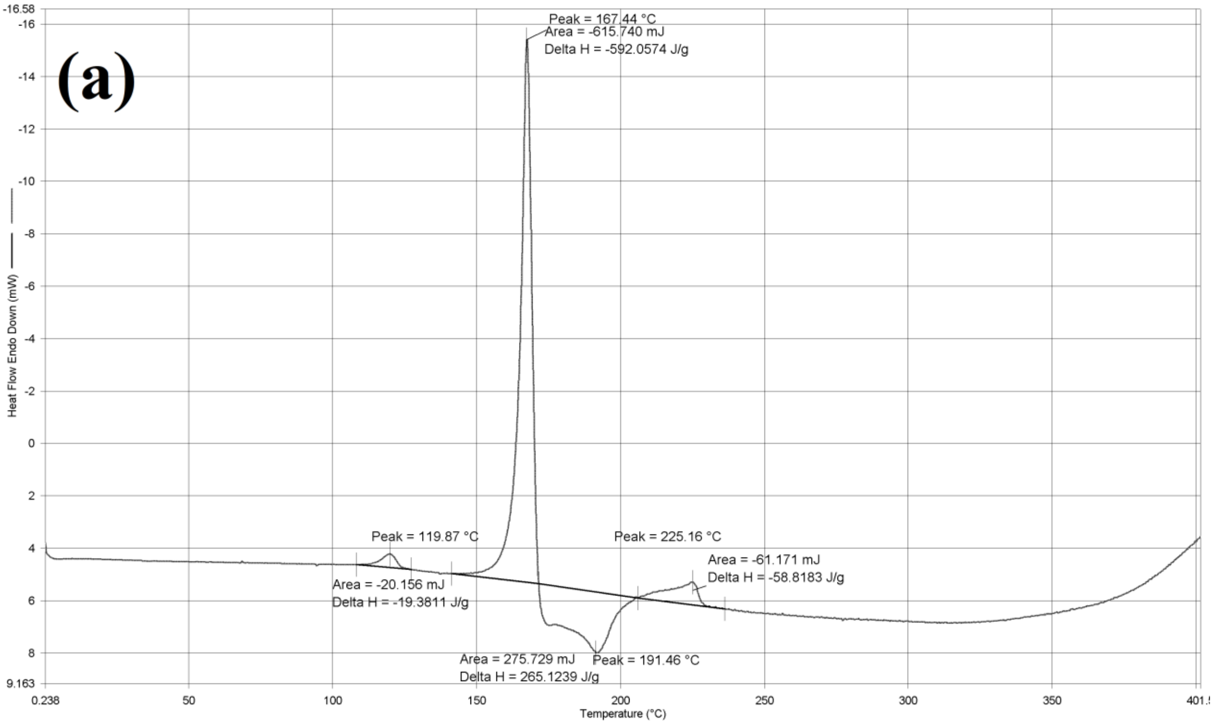

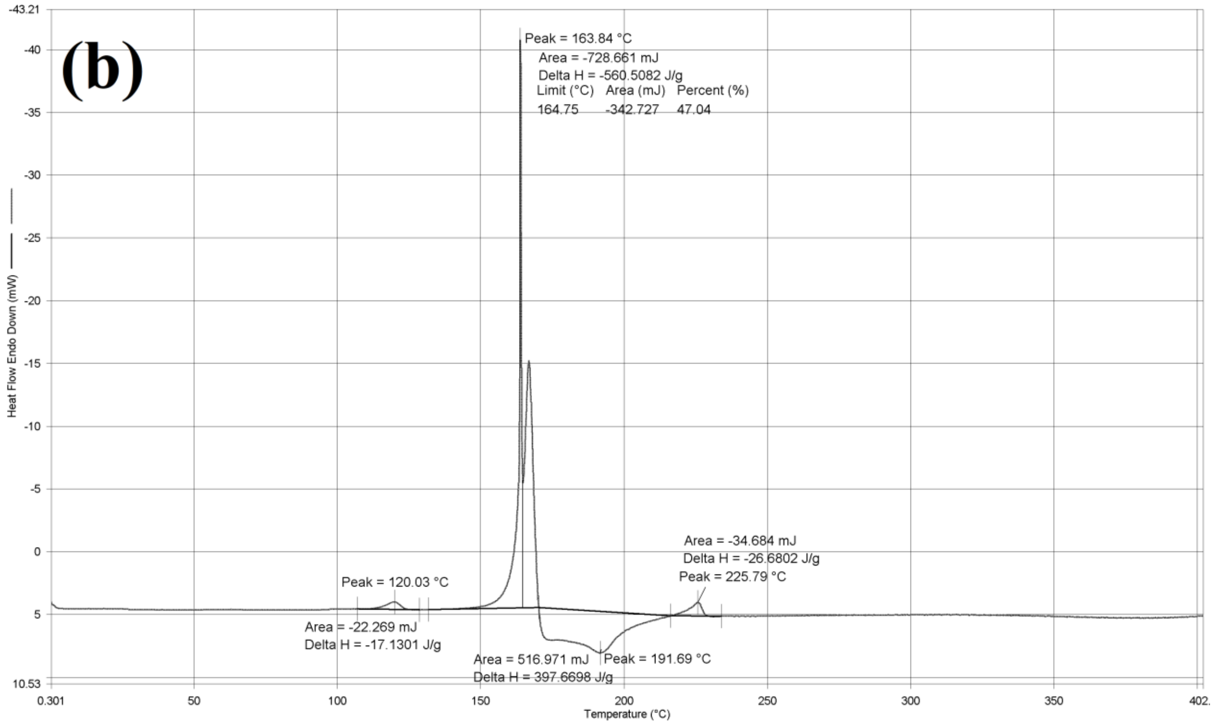

Figure S9. The DSC curves of compound **1** in (a) an oxidative and (b) an inert atmosphere.

Table S1. DSC results of compound **1** in an Ar and O_2 atmosphere at a 5 °C/min heating rate.

Peak temperature, °C	Reaction heat, kJ/mol	Peak temperature, °C	Reaction heat, kJ/mol
N_2		O_2	
120 .0	-8.719	119.9	-9.865
163.8	-285.293	167.4	-301.351
164.8	-174.445		
191.7	263.163	191.5	134.945
225.9	-13.580	225.6	-29.938

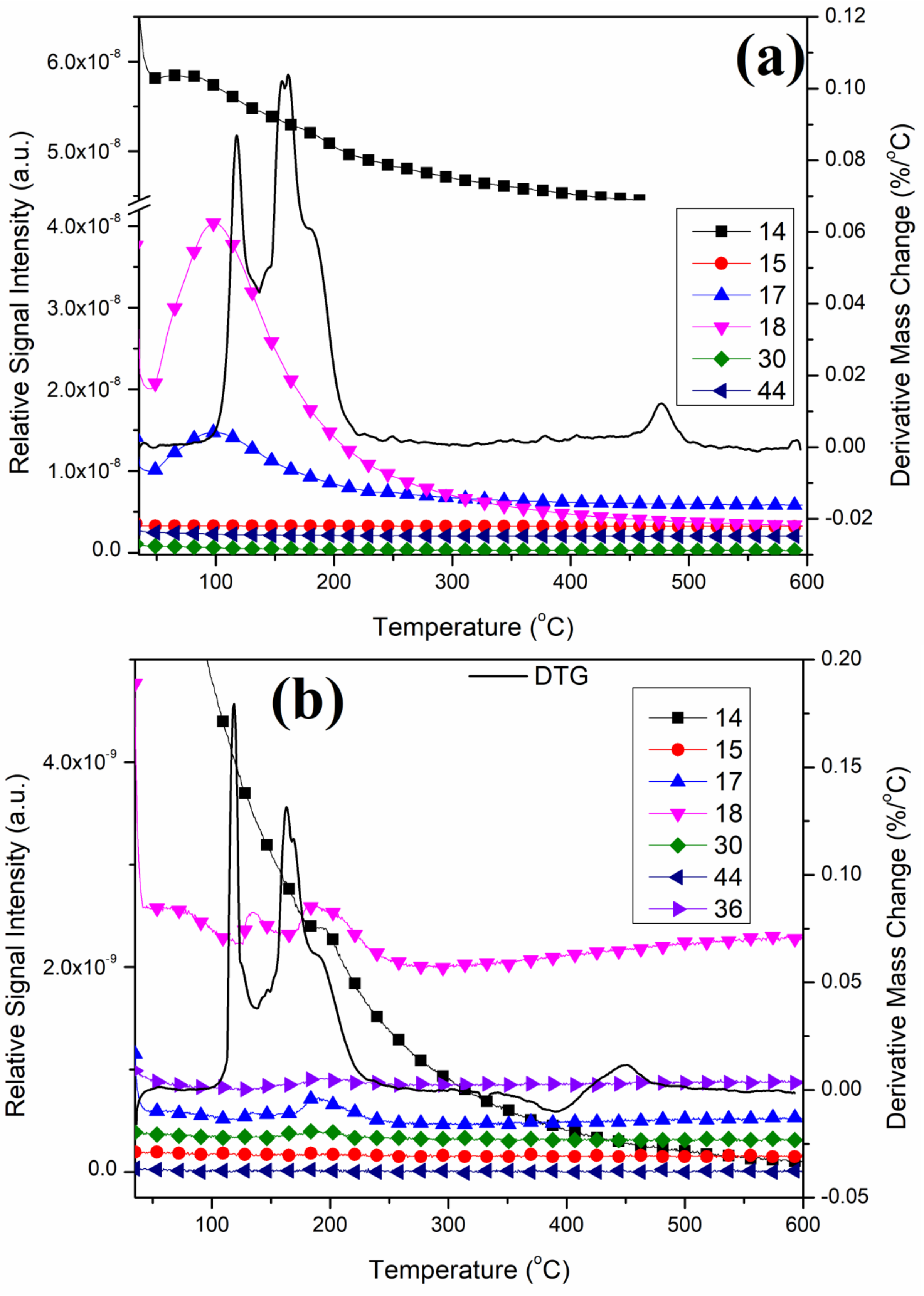

Figure S10. The TG-MS measurements of compound **1 (a)** in an oxidative and **(b)** in an inert atmosphere.

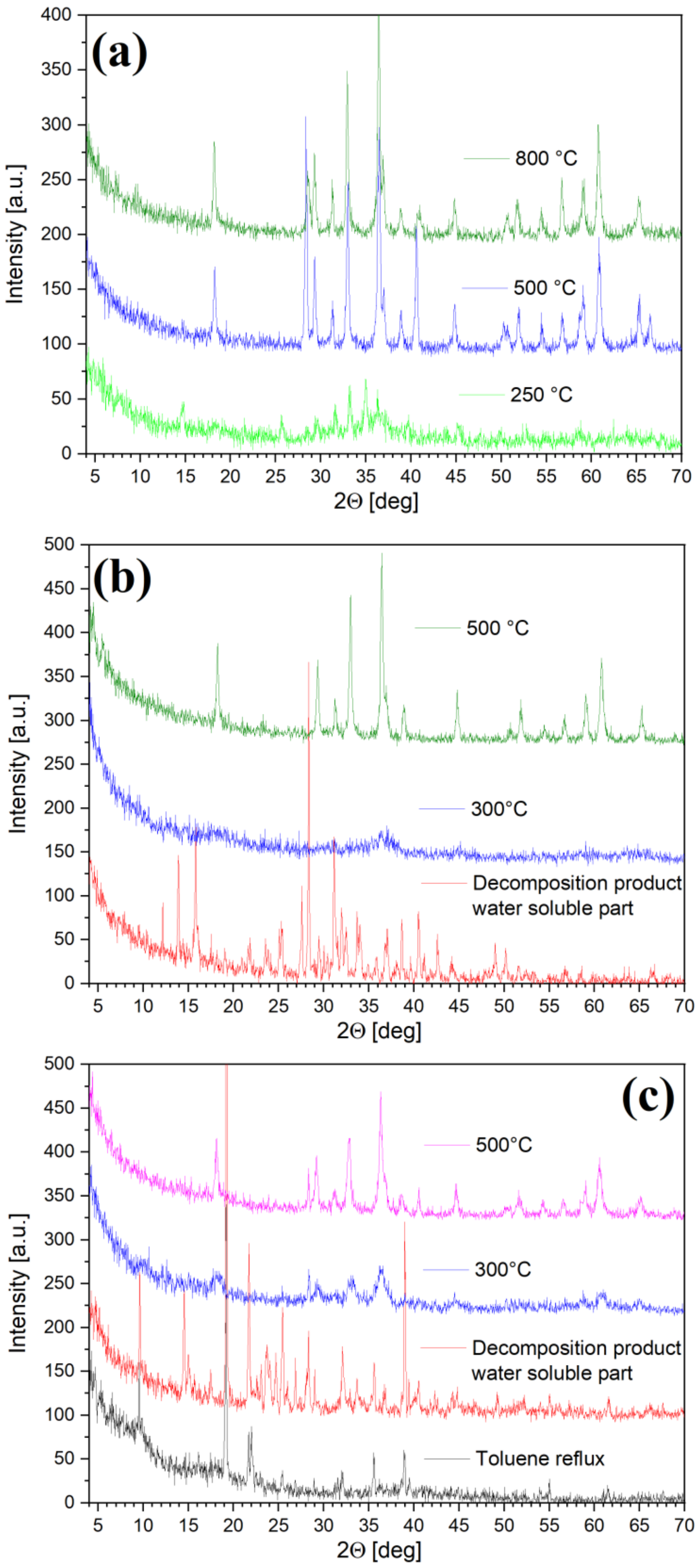

Figure S11. The PXRD of the decomposition products prepared from compound **1** (a) in an air atmosphere, (b) in an air atmosphere without the water-soluble part, and (c) in toluene at reflux temperature with subsequent heat treatment in a furnace in an air atmosphere.

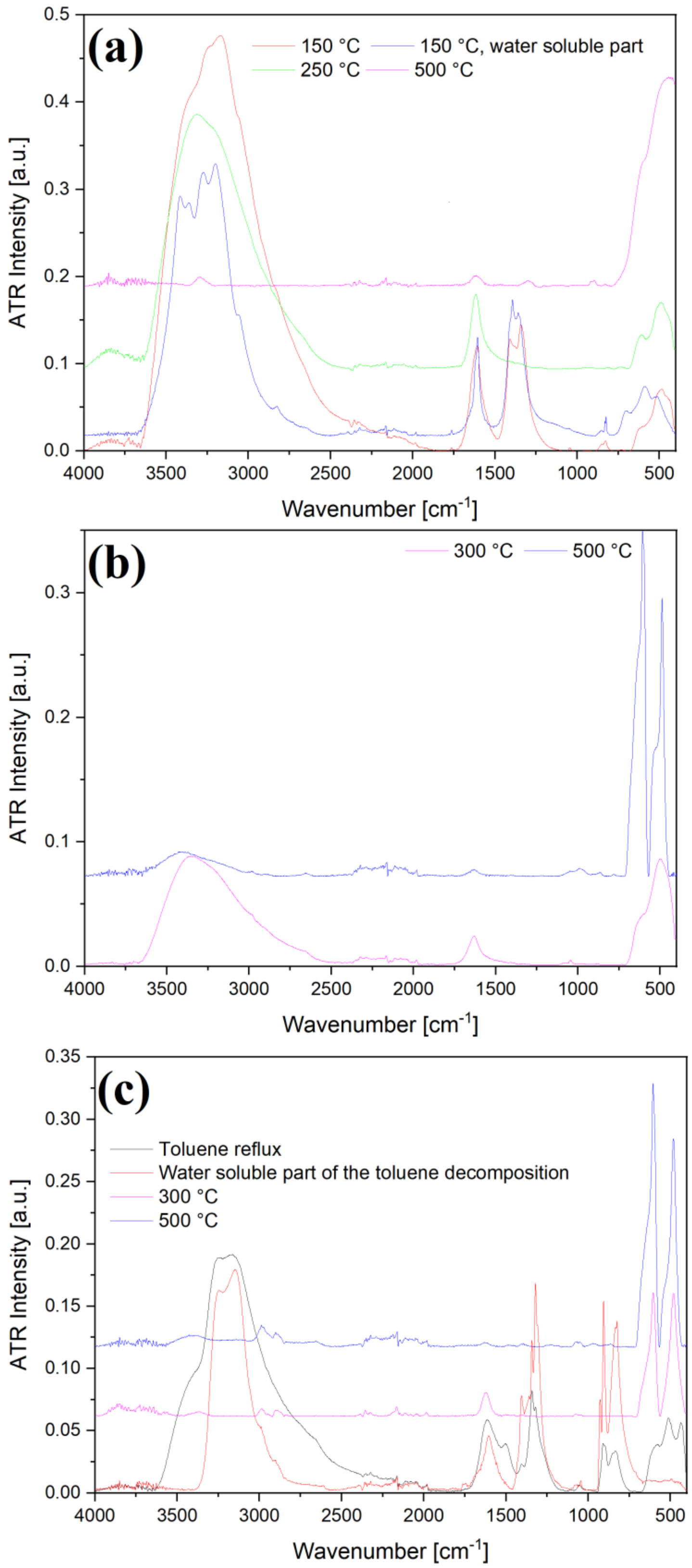

Figure S12. The IR spectra of the decomposition products prepared from compound **1** (a) in an air atmosphere, (b) in an air atmosphere without the water-soluble part, and (c) in toluene at reflux temperature with subsequent heat treatment in a furnace in an air atmosphere.
